# Pulmonary Sarcoidosis: Experimental Models and Perspectives of Molecular Diagnostics Using Quantum Dots

**DOI:** 10.3390/ijms241411267

**Published:** 2023-07-10

**Authors:** Natalia Linkova, Anastasiia Diatlova, Yulia Zinchenko, Anastasiia Kornilova, Petr Snetkov, Svetlana Morozkina, Dmitrii Medvedev, Alexandr Krasichkov, Victoria Polyakova, Piotr Yablonskiy

**Affiliations:** 1 St. Petersburg Research Institute of Phthisiopulmonology, Ligovskii Prospect, 2-4, 191036 Saint Petersburg, Russia; me@diatlova.ru (A.D.); ulia-zinchenko@yandex.ru (Y.Z.); info@spbniif.ru (A.K.); snetkov.pp@gmail.com (P.S.); i_norik@mail.ru (S.M.); vopol@yandex.ru (V.P.); piotr_yablonskii@mail.ru (P.Y.); 2 St. Petersburg Institute of Bioregulation and Gerontology, Dynamo pr., 3, 197110 Saint Petersburg, Russia; rsc-ide@yandex.ru; 3 Chemical Bioengineering Center, ITMO University, Kronverksky Pr, 49A, 197101 Saint Petersburg, Russia; 4 Department of Radio Engineering Systems, Electrotechnical University “LETI”, Prof. Popova Street 5F, 197022 Saint Petersburg, Russia; krass33@mail.ru; 5 Department of Hospital Surgery of the Faculty of Medicine, St. Petersburg State University, University Embankment, 7-9, 199034 Saint Petersburg, Russia

**Keywords:** sarcoidosis, quantum dots, molecular diagnostics, experimental models of sarcoidosis

## Abstract

Sarcoidosis is a complex inflammatory multisystem disease of unknown etiology that is characterised by epithelioid cell granulomatous lesions affecting various organs, mainly the lungs. In general, sarcoidosis is asymptomatic, but some cases result in severe complications and organ failure. So far, no accurate and validated modelling for clinical and pathohistological manifestations of sarcoidosis is suggested. Moreover, knowledge about disease-specific diagnostic markers for sarcoidosis is scarce. For instance, pulmonary granulomatosis is associated with the upregulated production of proinflammatory molecules: TNF-α, IL-6, CXCL1, CCL2, CCL18, CD163, serum angiotensin-converting enzyme (sACE), lysozyme, neopterin, and serum amyloid A (SAA). Quantum dots (QDs) are widely applied for molecular diagnostics of various diseases. QDs are semiconductor nanoparticles of a few nanometres in size, made from ZnS, CdS, ZnSe, etc., with unique physical and chemical properties that are useful for the labelling and detection in biological experiments. QDs can conjugate with various antibodies or oligonucleotides, allowing for high-sensitivity detection of various targets in organs and cells. Our review describes existing experimental models for sarcoidosis (in vitro, in vivo, and in silico), their advantages and restrictions, as well as the physical properties of quantum dots and their potential applications in the molecular diagnostics of sarcoidosis. The most promising experimental models include mice with TSC2 deletion and an in silico multiscale computational model of sarcoidosis (SarcoidSim), developed using transcriptomics and flow cytometry of human sarcoid biopsies. Both models are most efficient to test different candidate drugs for sarcoidosis.

## 1. Introduction

Sarcoidosis is a multisystem inflammatory disease of unclear etiology, most often affecting the lungs (up to 90% of cases) and lymph nodes. Sarcoidosis is characterised by the formation of non-caseous granulomas and ranks first in frequency among granulomatous diseases. Women, as well as workers disproportionately exposed to chemical agents (metallurgical industry, agriculture, etc.), are at the highest risk of developing sarcoidosis [1,2]. Sarcoidosis is often asymptomatic for a long time or manifests with nonspecific symptoms (weakness, fatigue, and fever). The most severe complication of pulmonary sarcoidosis is an acute respiratory failure at advanced disease stages, when granulomas eventually become fibrotic tissue [3]. In most cases, diagnostics include histological verification to exclude alternative conditions (tuberculosis, fungal lesion, etc.) [4].

Molecular mechanisms behind the development of sarcoidosis are not definitively established. Its pathogenesis is associated with infection-related, genetic, and immune factors. Infection-related factors include microbial agents, such as *Mycobacteria* and *Propionibacteria*, as a likely cause of sarcoidosis. This hypothesis is supported by the meta-analyses conducted from 1980 to 2015, confirming the link between sarcoidosis and mycobacteria (*M. tuberculosis*, non-tuberculosis strains) [5,6]. In addition, some patients with sarcoidosis revealed high plasma levels of antimycobacterial antibodies to HSP70 heat shock protein [7]. A CD4+ T-cell immune response to mycobacterial katG antigens, Early Secret Antigenic Target 6 (ESAT 6), SodA, and heat shock proteins was present in bronchoalveolar lavage (BAL) fluid from patients with lung sarcoidosis [8]. A different opinion about the key role of *P. acnes* in the pathogenesis of sarcoidosis is no less widespread. Endogenous infection caused by symbiotic *P. acnes* can lead to the formation of granulomas in individuals who are predisposed to a hypersensitive Th1 immune response against an intracellular proliferation of latent *P. acnes* [9]. Exposure to metals and other inorganic substances can activate the immune response and eventually trigger sarcoidosis [2]. The most prominent model for the pathogenesis of sarcoidosis suggests aberrant innate and adaptive immune responses against unidentified inciting antigens in genetically predisposed individuals [10]. Some HLA, MHC2TA, BTNL2, CCR2, CCR5, TNF, and ANXA gene polymorphisms are associated with an acute presentation of sarcoidosis—Lofgren’s syndrome [11,12], whereas other polymorphisms in these genes are implicated in non-Lofgren’s syndrome. Moreover, some HLA and CCR5 polymorphisms reduce the risk of sarcoidosis, such as the CCR2-64I polymorphism in the Japanese population [13].

In this regard, the analysis of existing models for sarcoidosis as well as the development of novel disease models are critical to elucidate the disease pathogenesis and to advance diagnostics and therapies [14]. This review is an attempt to analyse sarcoidosis models and disease-specific molecular markers, using the quantum dots technique.

## 2. Experimental Models of Sarcoidosis

Based on the available data on the pathogenesis of sarcoidosis, various in silico, in vitro, and in vivo granulomatous disease models were developed. Sarcoidosis models come in 5 types: BAL cell-based models, human peripheral blood mononuclear (PBMCs) models, investigation of sarcoid biopsies, animal models, and computational models. According to Bernard V. et al., a good model should meet the following criteria: to be isomorphic, homologous, and predictive [15].

### 2.1. BAL Cell-Based Models

Investigations of BAL cells from patients with sarcoidosis are considered as one of the ways to study the molecular mechanisms of the pathogenesis. The method consists in the BAL procedure, extracting viable cells from the obtained liquid and seeding them on a culture medium. Flow cytometry is used to sort various types of cells, for example, CD4+, CD8+ lymphocytes, or macrophages. Using this method Wiken et al. (2010) demonstrated the absence of the differences between M1/M2 polarisation of alveolar macrophages in patients with and without sarcoidosis. In the same study, the expression of toll-like TLR-2 mRNA in BAL cell culture was reduced 2-fold in patients with sarcoidosis and reduced 4-fold in Lofgren’s syndrome compared to healthy controls [16].

In addition, it is possible to study BAL cells by immunocytochemistry [17]. Phenotyping of M1 and M2 alveolar macrophages was performed using antibodies for CD40 and CD163, respectively. It was demonstrated significant differences in the greater frequency of the M1 phenotype in BAL cells from patients with sarcoidosis compared to patients who suffered from other interstitial lung diseases (CD40+++ 61% vs. 49%). The proportion of CD163- was significantly higher in the SA group (5% vs. 1%, *p* < 0.05). The proportion of CD163+ cells was elevated in the group of ILD, but without significance (43.6% vs. 34.6%). At the same time, there was no predominance of M1 or M2 macrophage phenotype among BAL cells obtained from patients with interstitial lung diseases: hypersensitive pneumonitis, nonspecific interstitial pneumonia, and idiopathic pulmonary fibrosis [17].

In sarcoidosis, BAL cells with the expression of the TWIST1 transcription factor—which is a gene associated with the macrophage phenotype—is a 5,1-fold increase comparing healthy donors. The BAL obtained from patients with sarcoidosis demonstrated relative mRNA expression of IFNγ (7-fold), IL18R1 (5-fold), IL12Rb2 (6-fold), STAT1 (2-fold), STAT4 (3-fold), CXCL11 (4-fold), CXCL10 (3-fold), CXCL9 (5-fold), and CCL5 (6-fold), associated with M1 macrophage polarisation. TWIST1 protein synthesis in BAL cells increased with the addition of lipopolysaccharide (LPS) and TNF-α, according to the immunofluorescence assay (quantitative data were not represented) [18].

It is believed that bone marrow mesenchymal stromal cells (CM-MSCs) can “reprogram” various types of macrophages for an anti-inflammatory phenotype. BAL cells (70–94% of macrophages) from patients with sarcoidosis and healthy donors were cultured with CM-MSCs and stimulated LPS. After that, the content of IL-10 and TNF-α in cultures was determined. In cells obtained from patients with sarcoidosis, a 20-fold decrease in the concentration of TNF-α—which characterises the pro-inflammatory phenotype M1—and a 50-fold increase in the concentration of IL-10—a marker of the anti-inflammatory phenotype M2—were revealed in comparison with cultures without adding BM-MSCs. BAL from healthy donors did not demonstrate changes of cytokine synthesis during the addition of BM-MSCs [19].

The TLRs are a family of immune recognition molecules that initiate the production of proinflammatory cytokines in response to the molecular patterns of pathogens. Cytokines TNF-α and IL-6 are possibly involved in the granuloma formation. Gabrilovich et al. (2013) showed that the synthesis of proinflammatory cytokines TNF-α and IL-6 increased in BAL cells in response to the TLR-2—lipoprotein M. tuberculosis (LpqH) stimulation. Stimulation of these LPS cells did not affect the production of cytokines by BAL cells in sarcoidosis. TLR-2 is capable of forming heterodimers with TLR-1 and TLR-6: TLR-2/1 and TLR-2/6, respectively. To characterise the mechanisms of TLR-2 hypersensitivity in sarcoidosis, the response of BAL cells to ligand stimulation of these heterodimers was analysed. For TLR-2/1, such a ligand is the lipopeptide Pam-3-Cys-SKKKK (Pam-3-Cys). For TLR-2/6, the peptide FSL-1 is obtained from the lipoprotein M. salivarum LP44 (MALP). Stimulation of BAL cells obtained from patients with sarcoidosis with Pam-3-Cys did not affect cytokine synthesis. With the action of FSL-1 in sarcoidosis cells compared with healthy donors, a 2-fold decrease in the concentration of TNF-α was observed. Probably, in pulmonary sarcoidosis, there is a violation of the regulation of the local heterodimeric TLR-2 response [20].

Kraaijvanger et al. (2020) proposed a comprehensive review of blood serum and BAL fluid biomarkers produced by the innate and adaptive immunity cells involved in the formation of granulomas in sarcoidosis. According to this model, sarcoidotic granulomas consist of a dense nucleus formed by epithelioid and multinucleated giant cells, and they are surrounded by T-helpers, B-lymphocytes, macrophages, and dendritic cells. During granuloma formation, these immune cells release numerous signalling molecules. Macrophages play a key role in the formation of granulomas by producing angiotensin converting enzyme (sACE), lysozyme, neopterin, CD163, CCL18, and serum amyloid A (SAA). In addition, through antigen presentation, macrophages induce T-cell activation and upregulated expression of the soluble IL-2 receptor (sIL-2R). The BAFF factor is involved in B cell activation and, thus, contributes to granuloma formation [21].

### 2.2. Combination of BAL Cell-Based Models and Computational Analysis Methods

Comparison of gene expression in sarcoidotic BAL cells revealed more than 1500 differentially expressed genes in contrast to healthy subjects [22]. For example, in patients with sarcoidosis, the expression of genes responsible for T-cell signalling, lymphocyte activation, and proliferation was increased, while the expression of genes regulating signal transduction, intercellular interaction, and antiviral protection was downregulated. For a more comprehensive investigation of the sarcoidotic transcription profile, the computational method of functional enrichment analysis (Gene set enrichment analysis, GSEA) was used. This method is designed to identify sets of genes that may be associated with disease phenotypes. Patients with sarcoidosis and healthy controls reported differences in the expression of 83 sets of genes. The products of these genes were involved in signalling pathways associated with adaptive immunity, T-cell signalling, graft-versus-host disease development, as well as IL-12, -23, -17 signalling. In addition, patients with sarcoidosis revealed activation of inducible PSMB8, PSMB9, and PSMB10 catalytic proteasome subunits. This suggests that sarcoidosis is associated with a proteolytic environment and substrate degradation. This study is an attempt to combine BAL cell-based models and computational methods of molecular genetics.

### 2.3. Investigation of Peripheral Blood Mononuclears (PBMC)

Recently, a model with PBMC exposed to purified protein derivatives (PPD) of *M. tuberculosis* immobilized on polystyrene granules was developed. After 7 days, granuloma-like multicellular aggregates were formed in the culture with the presence of macrophages in the centre and lymphocytes on the periphery. In the sarcoidosis and healthy control PBMC, the incubation with PPD granules activated the Th1-immune response. But, in the culture of PBMC from patients with sarcoidosis, the concentration of IFN—γ was less markedly increased 3,5-fold, and the concentration of TNF-α was more pronounced (4-fold) compared to the control cultures. It is obvious that granules coated with the human serum albumin (HSA) caused selective reaction of monocytes/macrophages (GM-CSF, TNF-α) in PBMC in sarcoidosis, not in the control group. Such nonspecific activation of macrophages in the response to HSA-coated granules indicates the activation of macrophages and T cells in patients with pulmonary sarcoidosis [4].

The same model was used to demonstrate that macrophages retain the M2 phenotype in sarcoidotic PBMC cultures. This phenotype was determined by gene expression and CD163 protein receptor immuno-staining in PPD granules culture, in contrast to uncoated granules. In addition, analysis of the mRNA expression profile demonstrated that 1274 differentially expressed genes were detected in PBMCs obtained from patients with sarcoidosis after PPD stimulation. In a group of patients with sarcoidosis, IL-13 cytokine was reported to regulate differential gene expression. M2 polarisation of macrophages was probably caused by the STAT6 factor activation by IL-13 [23].

A modification of the described model suggests using microparticles obtained from the cell wall of Mycobacterium abscessus (MAB), instead of PPD granules, for PBMC culture. The granuloma is observed after 72 h of exposure. MAB-stimulated granulomas have Th1 and Th17 inflammatory profiles dominated by IL-7 (2-fold), IL-7R (2-fold), and IFN-γ expression (1,4-fold). Notably, when exposed to α-melanocyte stimulating hormone (𝛼-MSH), the model produced an anti-inflammatory effect with reduced IL-7, IL-7R, and IFN-γ expression. This effect is linked to the CREB transcription factor activation [24]. Here, we suppose that this modification is of critical relevance, since non-tuberculosis mycobacteria are more commonly found in a variety of environmental habitats rather than *M. tuberculosis*, while the immunophenotype of their granulomas is similar to that of clinical sarcoidosis. Comparison of PBMCs’ transcriptomes in sarcoidosis and in healthy controls revealed 270 differentially expressed genes involved in IL-1, IL-6, and IL-8 signalling pathways; mononuclear migration; and regulation of cellular LPS response [25]. In general, PBMC-based models demonstrate some advantages of an in vitro granuloma model, including 3D cell culture and granule-like structures producing Th-1 cytokines. Under pathogen stimulation, PBMC models also reproduce genetic expression patterns.

The newest class of in vitro sarcoidosis models is based on pathogenic environmental factors, associated with the release of carbon nanoparticles into the ambient environment, such as fuel combustion, industrial manufacturing, or burning fires. Individuals exposed to such nanoparticles are at risk of developing chronic lung diseases, including sarcoidosis and fibrosis [26]. An experimental in vitro model of sarcoidosis was developed using PBMCs exposed to multi-walled carbon nanotubes (MWCNT). On day 7 of incubation with 0.25 mg/mL of MWCNT, PBMCs exhibited organised clusters of highly vacuolized CD68+ macrophages surrounded by CD3+ T cells. The number of such clusters was 4-fold higher than in non-MWCNT cultures, and the area of the clusters was 2,5-fold higher compared to control. MWCNT-challenged PBMCs demonstrated elevated expression of TNFa, IL-6, and IL-10 proinflammatory cytokines. By day 7 of MWCNT incubation, the number of differentially expressed genes increased by almost 4 times compared to day 1 of incubation (802 versus 226 genes). Most of the genes were associated with immune defence mechanisms, chemotaxis, cell mobility, and migration [27]. It is noteworthy that the model is unique for its pioneering attempt to broaden the application of carbon nanotubes beyond modelling granulomatous inflammation in animals. This model is promising, considering the availability of biologic material and no requirement to use pathogenic microorganisms.

**Figure 1:**
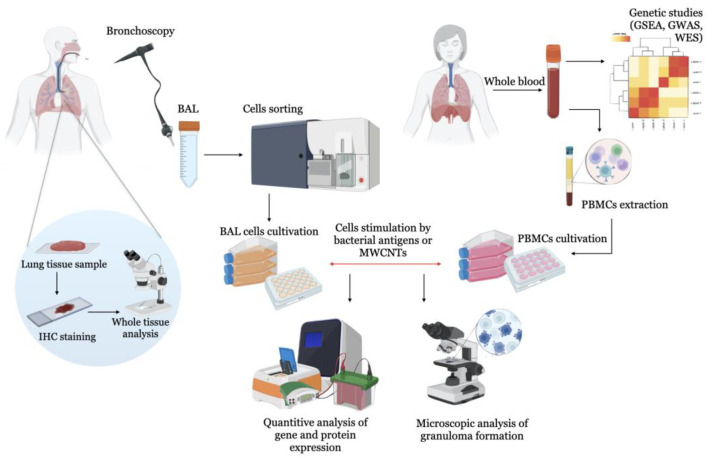
Schematic representation of the in vitro sarcoidosis model to study human lung cells. Cells are isolated from lung tissue biopsy, BAL, or blood of sarcoidotic patients and healthy donors. In healthy controls without pulmonary pathology, bacterial agents (Mycobacterium protein particles, *P. acnes*) or carbon nanoparticles (MWNCTs) are added to initiate granulomas and pulmonary fibrosis as major signs of sarcoidosis.

The scheme of the use of human cells in the sarcoidosis modelling is in Figure 1.

### 2.4. Animal and Experimental Models of Sarcoidosis

Animals, except horses, do not develop sarcoidosis, which complicates the development of experimental animal models of the disease [23,28]. Currently, there is no commonly accepted animal model of sarcoidosis; however, a few techniques are used to reproduce the pathohistological signs of granulomatous diseases.

One of the approaches is preliminary sensitisation of rodents to candidate antigens, followed by their intrapulmonary administration. Lewis rats and C57BL/6 mice were immunised using intraperitoneal injection of either *M. tuberculosis* whole cell lysate in complete Freund’s adjuvant or recombinant mKatG protein (*Mycobacterium tuberculosis* catalase–peroxidase) in incomplete Freund’s adjuvant. Two weeks later, the animals were injected with mKatG-coated sepharose granules via intratracheal instillation. Granulomatous response to mKatG granules was associated with the proliferation of antigen-specific T cells and the production of IFN-γ. Chen et al. (2010) used this model to demonstrate that chronic exposure to SAA exacerbates persistent experimental granulomatous inflammation in animals with prior mKatG sensitisation, which is probably triggered by TLR-2 [29]. The model, however, has suspicious specificity to sarcoidosis due to the use of *M. tuberculosis* and its antigens to induce granuloma.

Despite this limitation, a similar model of sarcoidosis was proposed in the C57BL/6 line mice, which were previously sensitised by injection of the incomplete Freund adjuvant with *Mycobacterium* sodA (superoxide dismutase A) and sodA-coated granules. Granulomas concentrated around the granules, and an increased CD4+ immune response and elevated levels of IL-2 and IFN-γ in the BAL were detected in such animals (9-fold and 12-fold, respectively, compared to control BAL obtained from mice without sodA challenge [30]. However, there is a question about the specificity of this model.

Alongside with Mycobacterium, *P. acnes* is also used as a sensitising agent in the sarcoidosis models. Kishi et al. (2011) sensitised C57BL/6 mice using *P. acnes* lysate with incomplete Freund adjuvant and simultaneous administration of dendritic cells two times, with a two-week timespan between sensitisations. After a week, granulomas were identified in the lung. If dendritic cells were incubated with *P. acnes* prior to administration, granulomas were larger in size and number. The advantage of this model is explained by significant similarity in cellular composition of granulomas and sarcoidotic granulomas exhibiting lymphocytes, epithelioid cells, and multinucleated giant cells. In BAL cells from animals, the levels of CXCL9 and CXCL10 as well as Th1-expressed cytokines and chemokines, including TNF-α and IFN-γ, were increased [31]. A different sarcoidosis model proposed to immunise C57BL/6 mice by administering three subcutaneous injections of 400 mcg of heat-killed *P. acnes* with complete Freund adjuvant at a two-week interval. Next, CD4+ T cells were isolated from the lymph nodes of immunised animals and injected into the tail vein of mice without immunisation. Two weeks after intravenous administration of T cells in mice, there was an aggregation of epithelioid and mononuclear cells and a 2-fold increase in ratio of CD4+/CD8+ observed in the BAL and peribronchial lymph nodes of mice with challenge of *P. acnes* in CFA comparing mice with only CFA administration. The increased number of CD4+ T lymphocytes expressed IFN-γ, which is similar to the histopathological characteristics in sarcoidosis [32].

Granulomas were predominantly located in subpleural, peribronchial, and perivascular regions of the lungs and featured antigen-presenting cells in the centre and CD4+ T lymphocytes in the periphery. CD4+ T cells expressed IFN-γ but not IL-4, while the CD4+ to CD8+ lymphocyte ratio was increased in BAL. This *P. acnes* immunisation model is compatible with the lung histopathology in patients with sarcoidosis [32].

Werner et al. (2017) revealed that injection of *P. acnes* isolated from sarcoidosis patients, instead of heat-killed P. acnes, promotes formation of larger granulomas. Interestingly, mice with mutations in the Myd88 and Cyb genes showed increased *P. acnes* persistence in the lungs [33].

Barna et al. investigated the role of MWCNT carbon nanoparticles in mice with a deficiency of genetic factors associated with human sarcoidosis. This may contribute to the development of immunoassociated lung granuloma [34]. In mice with the PPARγ (a receptor activated by the peroxisomal proliferator γ) knockout, the instillation of MWCNT led to the formation of granulomas in 60 days [35]. In wild-type mice exposed to MWCNT, an increase in the expression of osteopontin and CCL2 and the proinflammatory factor NF–kB was found, which decreased with the introduction of rosiglitazone, a specific ligand PPARγ that promotes its activation [36].

The deficiency of ABC-transporter of alveolar macrophages ABCG1 is also associated with granulomatous inflammation in the lungs [37]. Relative mRNA expression of ABCG1 was decreased 2-fold in the alveolar macrophages of wild-type mice and animals with PPARγ knockout 60 days after the instillation of MWCNT [38]. The increased expression of the MMP12 gene and MMP12 protein was found in sarcoidosis. An increase in MMP12 synthesis correlated with the severity of the disease. Relative mRNA expression of MMP12 in patients with severe sarcoidosis was 11-fold higher, and protein level was 2-fold higher compared to healthy donors [39]. Wild-type mice showed an increase in Mmp12 expression from day 3 to day 60 after MWCNT administration. Relative mRNA expression of Mmp12 was 10-fold, 300-fold, 100-fold, and 45-fold higher in mice at 3rd, 10th, 20th, and 60th days after MWCNT administration compared to control with PBS administration. MMP12 was 4,5-fold elevated in MWCNT-instilled PPARγ KO BAL cells compared to PBS-instilled PPARγ KO BAL cells. Mmp12 gene expression in PPARγ KO mice was intrinsically increased 20-fold and further increased after MWCNT instillation compared to C57BL/6. In BAL cells of animals with Mmp12 knockout, a 1,5-fold increase in the expression of PPARγ was demonstrated. The level of IFN-γ expression during Mmp12 knockout stays as MWCNT-instilled [40]. Thus, the use of the MWCNT model allowed the establishment of the role of PPARγ, ABCA1, ABCG1, and MMP12 in the pathogenesis of granulomatous inflammation [41].

Thus, a large number of signalling molecules are involved in the pathogenesis of sarcoidosis, which may be markers of the disease. The main ones are listed in Table 1.

There is a variation of the MWCNT model, when ESAT-6, a peptide of 15 amino acid residues and a component of mycobacterial antigen, is injected into animals together with MWCNT. For 60 days after simultaneous administration of ESAT-6 peptide with MWCNT, an increase in granulomatous lesion and pulmonary fibrosis was revealed compared with the administration of MWCNT [42]. Increased fibronectin expression was observed in PPARγ knockout mice injected with a combination of ESAT-6 and MWCNT [43]. In wild-type mice, 60 days after the administration of ESAT-6 and MWCNT, there was no persistence of ESAT-6 in the lungs, while in mice with knockout PPARγ, ESAT-6 was detected. This confirms the hypothesis that the deficiency of PPARγ may contribute to the prolonged action of the mycobacterial antigenic peptide ESAT-6 on the body.

Genetic modifications in the study of sarcoidosis are used as an additional method, in the case of MWCNT for example, when their effects were studied in mice with knockout of various genes involved in the pathogenesis. The deletion of the Tsc2 gene makes it possible to obtain manifestations of sarcoidosis in mice. TCS2 (tuberin) is a tumour suppressor that normally suppresses the activation of the mTORC1 complex, a negative regulator of autophagy. Deletion of Tsc2 leads to the activation of mTORC1 and induced hypertrophy, proliferation, and granulomatous inflammation in mice in vivo. Macrophages with Tsc2 gene expression deficiency form granuloma-like structures [44]. However, studies using genome-wide association search (GWAS) have not revealed variations of the Tsc2 gene as risk factors for sarcoidosis. More recent studies using complete exome sequencing (WES) of familial cases of sarcoidosis have demonstrated the involvement of mTOR and autophagy in the pathogenesis of the disease [45,46,47,48,49].

**Figure 2:**
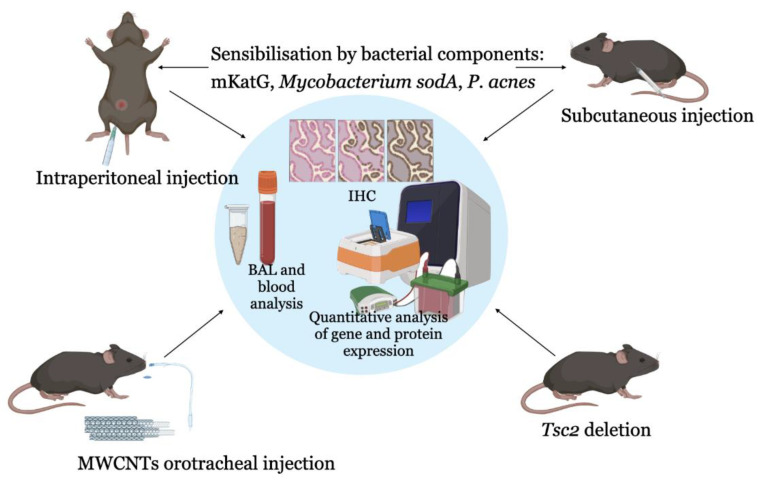
A scheme for the creation of sarcoidosis models in animals. For this purpose, animals are sensitised by preliminary administration of bacterial antigens (Mycobacterium protein particles, *P. acnes*) or carbon nanoparticles (MWNCTs), which initiate granulomas and pulmonary fibrosis as the main signs of sarcoidosis. Mice with deletion of the Tsc2 gene, a regulator of the mTOR signalling pathway, are also used to simulate sarcoidosis. Abbreviations: BAL—bronchoalveolar lavage, IHC—immunohistochemistry, PBMCs—peripheral blood mononuclear cells, GSEA—gene set enrichment analysis, GWAS—genome-wide association studies, WES—whole-exome sequencing, MWNCTs—multi-walled carbon nanotubes.

Thus, the most used common models of granulomatous lung lesions in animals include intraperitoneal or subcutaneous sensitisation with bacterial components, as well as the introduction of foreign particles—multi-walled carbon nanotubes, which is a reflection of the bacterial theory of sarcoidosis and the theory of immune response to environmental components (Figure 2). In addition, deletion in the Tsc2 gene in mice is also becoming widespread among in vivo sarcoidosis models.

### 2.5. Computational Models of Sarcoidosis

Based on stochastic Petri nets [50], a two-component computer simulation in silico SarcoidSim is being developed, which simulates intracellular signalling pathways mTOR and MAPK in macrophages involved in the pathogenesis of sarcoidosis. Such a model will allow an evaluation of the interaction of drugs and immune cells in sarcoidosis [51]. Previously, a model was developed based on clinical data on cytokine levels in normal lung tissues and lung tissues in sarcoidosis. The effect of infliximab, anti-IL-12, anti-IFN-ɣ, and TGF-β on the granuloma radius was studied by this model. The obtained data showed that these substances could slow down the development of granulomatosis. Thus, this model can be used to predict the treatment effectiveness [52].

Mathematical modelling can be used to evaluate the interactions of immune cells and inflammatory mediators during granuloma formation. The targets of therapeutic agents identified in this way can be tested in in vitro and in vivo models.

**Figure 3:**
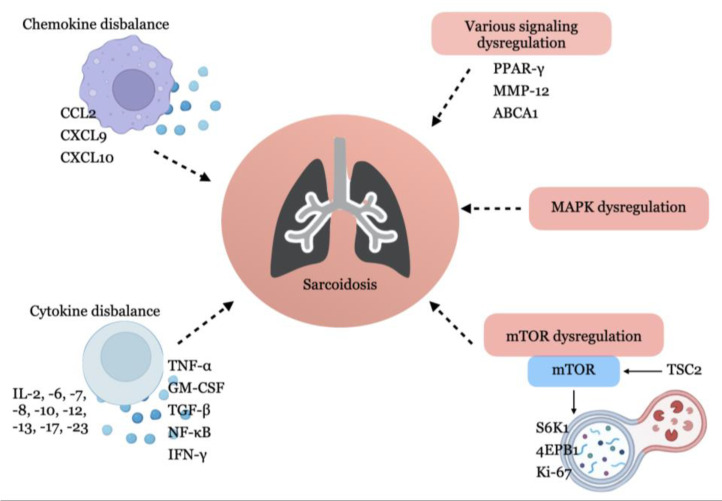
The main signalling molecules and pathways involved in the pathogenesis of sarcoidosis.

The summary of gene expression and protein synthesis used in experimental modelling of sarcoidosis is in Figure 3.

## 3. The Prerequisites for the Diagnosis of Sarcoidosis with Quantum Dots

Diagnosis of sarcoidosis is difficult due to the lack of uniform criteria and specific biological markers of the disease. The diagnosis is based on computed tomography data and biopsy of the affected tissues; one of the conditions is the exclusion of alternative causes with examination for tuberculosis, fungal infections, etc. [4]. A long diagnostic procedure and a high frequency of diagnostic errors requires the development of faster and more accurate diagnostic methods, remaining an actual task of modern biomedicine.

Such methods include the visualisation of tissues and organs with the registration of autofluorescence or specific staining of cells and tissues with contrasting agents [53]. Substances used for fluorescence diagnostics must have a number of properties: biocompatibility, photostability, sufficient brightness, high quantum yield, and the ability to conjugate with various guiding molecules for targeted binding.

Quantum dots (QDs) are a class of fluorophores, which are semiconductor nanocrystals with a size of 1–15 nm. According to the chemical composition, 12 classes of QDs are distinguished in accordance with the position of their constituent elements in the Periodic Table of Mendeleev [54]. Classification with the type of structure includes having a nuclear core and shell and doped QDs. QD cores consist of elements in groups II–VI or III–V of the periodic table. Examples of QDs in groups III–IV are indium phosphide (InP), indium arsenide (InAs), gallium arsenate (GaAs), and gallium nitride (GaN), and groups II–VI include zinc sulfide (ZnS), zinc–selenium (ZnSe), cadmium–selenium (CdSe), cadmium–tellurium (CdTe), or combinations of elements [55].

Due to small size, QDs obey the laws of quantum mechanics and have special physicochemical properties and optical characteristics that distinguish them from other fluorophores used in biomedical research. QDs have a high molar extinction coefficient, a wide absorption spectrum, a narrow and symmetrical fluorescence spectrum, and a high resistance to photodegradation. A wide range of excitation makes it possible to use a mixture of several QDs in the study. The physicochemical properties of QDs depend on the method of their synthesis, which can be divided into four groups: colloidal synthesis, synthesis based on biomatrix, electrochemical assembly, and biogenic synthesis [54]. Dynamic light scattering (DLS), scanning, transmission, and atomic force microscopy are used to determine the size of QDs [56].

Biomedical applications of QDs currently include in vitro diagnostics, visualization of living cells, use in photodynamic therapy, and drug delivery.

### 3.1. In Vitro Diagnostics Using QDs

Due to the unique optical properties of QDs, it became possible to modify the method of immunohistochemistry (IHC). Standard chromogens that allow the visualisation of the immunohistochemical reaction have high photosensitivity and the ability of nonspecific binding, which can lead to the background staining of drugs. Some chromogens (for example, 3,3′-diaminobenzidine) have high toxicity and carcinogenicity [57,58]. The use of QDs has expanded the possibilities of using IHC. Fluorescent labelling of several molecules within a single sample was previously possible due to the use of fluorochromes linked to secondary antibodies. In 2001, the first IHC protocol using QDs was developed. Since then, a number of protocols for various tissues were published. The unique properties of quantum dots expand the possibilities of sensitivity of the method and quantitative evaluation of visualisation results. This allows for a more detailed analysis of the tissue structure, cellular localization, the relative content of antigens in the tissue, and their colocalization [59]. Several strategies based on non-covalent self-assembly have been described for the conjugation of proteins and other biomolecules to water-soluble QDs, which have found application in QD-IHC [60]. Xing et al. (2007) described protocols for the conjugation of antibodies with QDs, sample preparation of tissues for multichannel imaging and image processing, and their quantitative analysis. The duration of such a study takes from 2 to 4 days, depending on the number of biomarkers used [61]. QD-IHC can be combined with signal amplification methods, such as tyramide signal amplification (TSA), to further improve the sensitivity of the method [62].

A protocol for the prognostic diagnosis of triple-negative breast cancer was proposed based on the visualisation of the topoisomerase 2 alpha (TOP2A) marker using QDs [63]. Multiplex QD staining for biomarkers in clinical tissue samples increases the diagnostic potential of IHC and allows the detection of multiple markers on a single slide [64]. QD-IHC was used to visualise several antigens associated with prostate cancer for the reconstruction of malignant transformation of heterogeneous tissue [65]. Using QD-IHC, patterns of spatial and temporal coevolution of gastric and breast cancer cells and their surrounding stroma were demonstrated, tracking markers of integrity or destruction of the basement membrane, angiogenesis, and macrophage invasion [66].

Zhao et al. created “affibodic” QDS (AF-QDs) for the human epidermal growth factor receptor type 2 (HER2) in human lung tumour cells [67]. In addition, the QD-IHC method was combined with other methods, such as QD in situ hybridization (QD-ISH) to study RNA transcripts (the research used digital quantum dots Qdot605 and Qdot655, Quantum Dot Corp.; Hayward, California) [68,69].

Chen et al. (2009) evaluated the ability of QD-IHC to visualise caveolin-1 and PCNA in a lung cancer tissue micromatrix compared to traditional IHC. It was demonstrated that both methods revealed the studied markers in both maligned and normal lung tissue. However, the sensitivity of QD-IHC was higher than IHC. The detection level of caveolin-1 and PCNA using QD-IHC was 57% (40/70 samples) and 86% (60/70 samples), respectively, which was higher than the detection level of markers using IHC (47% (33/70 samples) and 77% (54/70 samples), respectively. Less pronounced background staining was observed with QD-IHC than with IHC [70].

Another type of semiconductor nanocrystals—rod-like semiconductor nanocrystals, QRs, injected intramuscularly into live mice—can be visualized using confocal microscopy in formalin-fixed tissue sections filled with paraffin. Despite the small number of QRs injected (25 picomoles), they were clearly visualised in muscle sections after 24 h, and their fluorescent signal was stronger than that of QDs CdSe/ZnS functionalised in a similar way. In addition, in the case of QRs, the signal on the slice was preserved even 21 days after injection. This indicates the need to apply various modifications of semiconductor nanocrystals depending on the type of experiment [71].

In addition to tissue staining, the use of QDs is possible in cell culture. Ornberg and Liu (2007) published a detailed protocol for the visualisation of nuclear, cytoplasmic, and membrane compartments in a number of cell cultures using commercial secondary antibodies conjugated with QDs. Various variants of fixation and permeabilization, as well as features of cell smear conclusion were described [72].

Taking into account the possibility of multichannel imaging, IHC with QD looks like a promising method for the study of the molecular aspects of the development of granulomatous inflammation in sarcoidosis; however, such studies have not yet been conducted.

### 3.2. In Vivo Cell Imaging

In addition, QDs are used to visualise cellular components. Due to their small size, QDs are captured by cells during incubation, allowing for visualisation using a fluorescent microscope. Once conjugated with tissue-specific ligand molecules, QDs can be used to visualise organs and tissues [59].

A number of works are devoted to the visualisation of lung tissue in inflammation, chronic obstructive pulmonary disease, and vascular lung pathologies in mouse models [73]. Saitoh Y et al. studied acute pulmonary hypertension in mice with the introduction of QDs associated with glutathione using cryotechnics [74]. The development of Qdc for use in infectious pathology continues. By in vivo labelling of Avian Influenza Virus using Qdc, a stable fluorescence intensity was detected in the mice lungs, and it was possible to visualise the viral infection noninvasively [75].

Using QDs, it is possible to modify the fluorescence-activated cell sorting (FACS) method, a flow cytofluorimetry technology used to evaluate the functioning of drug delivery systems, isolate individual cell populations, detect cell markers, and map immune cells [76]. For example, the use of QD CdTe as fluorescent probes conjugated with monoclonal antibodies against antigens A and B on the surface of erythrocytes for the study of blood groups using FACS is described. The resulting bioconjugate demonstrated high efficiency and was stable for 6 months [77]. QDs could potentially replace antibodies that are used to detect biomarkers. Unlike antibodies, QDs can be absorbed by cells, which makes it possible to stain not only molecules on the cell surface but also intracellular markers, and this eliminates the need for permeabilizing buffers that affect cell viability, fluorophore efficiency, and complicate experiments with FACS [78]. The possibility of the visualisation of cells and tissues using QDs at the body level in mice was demonstrated [79].

### 3.3. Prospectivities for the Use of Quantum Dots in the Diagnosis of Sarcoidosis

Quantum dots seem to be a promising method not only for targeted drug delivery but also for the diagnosis of various diseases. The combination of IHC and QDs may be a promising method for the diagnosis of sarcoidosis. Taking into account the fact that immune cells involved in the formation of granuloma in sarcoidosis form a certain secretory phenotype, it is possible to use IHC with QDs to simultaneously identify several biomarkers in samples of damaged tissue in order to more accurately identify the molecular and morphological characteristics of cells. The cytotoxicity of QDs is not a problem here, since the study is carried out on a fixed biomaterial, but it remains an invasive procedure, which may be a disadvantage of this method. However, in addition to the standard histological examination, the use of IHC with QDs can significantly increase the accuracy of differential diagnosis. It also seems possible to use a modification of the FACS method with QDs to sort BAL cells from patients with sarcoidosis [80].

Drobintseva et al. (2015) studied the cytotoxicity of some QDs and their effect on the morphology of breast carcinoma cells ZR-75-1 and normal human mononuclears. QD ZnSe:Mn/MPC (mercaptopropionic acid) did not affect cell morphology during 48 h of incubation, even in high concentrations (up to 17.5 mM/mL). However, Qds CdSe caused apoptosis of 20% of cells after 18 h of the incubation [81]. Thus, it is possible to use some QDs not only on a fixed biomaterial but also in the PBMCs model. On the other hand, there is evidence that QD705 with the Cd/Se/Te nucleus and ZnS shell causes adverse effects in the lungs of mice with intratracheal administration, stimulating tissue infiltration by neutrophils, granulomatous reaction, increased expression of cytokines, chemokines, and MMP12 genes. The modification of QD705 with PEG coating did not reduce these side effects [82].

Thus, the reduction of cytotoxicity of QDs and studies directed to their application in various experimental models of sarcoidosis are a promising area for the development of molecular medicine. It can be concluded that, despite the absence of a generally recognised model of sarcoidosis, studies of the occurrence and development of granulomatous inflammation in various models are being successfully conducted.

## 4. Conclusions

The search for optimal models of sarcoidosis is important for the diagnosis and development of effective therapy for this disease. Currently, five main models of sarcoidosis are described: BAL cell-based models, PBMC, studies of biopsies of damaged tissue, animal models, and computer models. Sarcoidosis modelling is carried out in vitro using human lung cells and in vivo in mice with the experimentally induced fibrosis and lung granulomas, as well as in animals with impaired expression of the Tcs2 gene—an activator of the mTOR signalling pathway. In most of the described models, it is possible to evaluate the expression of genes and synthesis of proteins involved in the pathogenesis of sarcoidosis.

The quantum dot method is promising for investigations of the molecular aspects of the granulomatous inflammation in sarcoidosis, however, such studies were not yet conducted. In addition, QDs have cytotoxic properties, which impose additional restrictions on this type of research.

This review indicates the need for the optimal combination of available methods for diagnosing and evaluating the molecular mechanisms of the pathogenesis of sarcoidosis in silico, in vitro, and in vivo. Such investigations are an actual task of molecular medicine.

## Figures and Tables

**Table 1:** Molecular markers in experimental models of sarcoidosis.

Experimental Model(s)	Marker	Function	Effects (Increased/Decreased)
BAL cells obtained from patients with sarcoidosis	TWIST1	Transcription factor associated with macrophage polarisation	increased [18]
	TNF-α	Pro-inflammatory cytokine	increased [21]
	CCL18	Chemokine	
	CD163	Scavenger–receptor, bacterial sensor	
BM-MSCs co-cultivated with BAL cells obtained from patients with sarcoidosis (anti-inflammatory “reprogramming” BAL cells)	TNF-α	Pro-inflammatory cytokine	decreased [19]
	IL-10	Anti-inflammatory cytokine	increased [19]
Serum obtained from patients with sarcoidosis	Lysozyme	Enzyme, serum marker of macrophage activation	increased [21]
	sACE	Enzyme, sarcoidosis marker	
	SAA	Acute phase protein	
	sIL-2R	Soluble cytokine receptor	
BAL cells stimulated by LPS or TNFa	TWIST1	Transcription factor associated with macrophage polarisation	increased [18]
PBMCs stimulated by MWCNTs	CD68	Monocyte-macrophages marker	increased [27]
	CD3	TCR co-receptor	
PBMC obtained from patients with sarcoidosis	IFN–γ	Pro-inflammatory cytokine	increased [4]
PBMCs stimulated by MAB microparticles	IL-7	Pro-inflammatory cytokine	increased, but decreased during α-MSH stimulation [24]
	IL-7R	Pro-inflammatory cytokine receptor	
	IFN-γ	Pro-inflammatory cytokine	
BAL obtained from C57BL/6 mice stimulated by *P. acnes* lysate	IFN-γ	Pro-inflammatory cytokine	increased [32]
BAL obtained from Mmp12-knocked out mice stimulated MWCNTs	IFN-γ	Pro-inflammatory cytokine	increased [41]
	PPARγ	Nuclear receptor protein that functions as transcription factor	increased [41]
BAL obtained from PPARγ-knocked out mice stimulated by *P. acnes* lysate	CCL2 (MCP-1)	Chemokine that regulates migration and infiltration of monocytes/macrophages	increased [36]
	Osteopontin	Extracellular matrix protein expressed by osteoblastic and mesenchymal stem cells	increased [36]
WT and PPARγ-knocked out mice stimulated by MWCNTs	MMP12	Matrix metalloprotease	increased [40]

## Data Availability

This is the review article. New data were not created.
